# 

**Published:** 1879-12

**Authors:** 


LUCIEN HOWE, M. D.

### CONTENTS:

PAGE.

ORIGINAL COMMUNICATIONS:

Pulmonary Diseases of Elevator Employees, by Prof. Thomas F. Rochester, M. D., 181
Chronic Purulent Inflammation of the Middle Ear, by Lucien Howe, M. D., . . 188

CLINICAL REPORTS:

Traumatic Aneurism, Clinical Remarks by C. C. F. Gay, M. D. Reported by Fred. Peterson, M. D., . . . . . . 199
Angular Curvature of Spine Treated by the Paper-and-Glue Brace. Reported by Bernard Bartow, M. D., . . . . . . 202
Perityphlitic Abscess, Treated with Aspiration through Rectum. Reported by E. D. Merriam, M. D., . . . . . . 205

TRANSLATIONS:

The Method used in Germany in the Treatment of Placenta Prævia. Translated by Louis Schade, M. D., . . . . . 206
Atrophy of Optic Nerve in Erysipelas of the Face, by Parinaud. Translated from the French by Lucien Howe, M. D., . . 207
Unanimo Consensu. Translated from the Danish by Herman Mynter, M. D., . 208
Cupro-Sulphate of Ammonia in Neuralgia of the Fifth Pair, by Fereol. Translated from the French by Lucien Howe, M. D., 209

SELECTIONS:

Remarks on Twenty-Five Cases of Splenotomy, . . . . . . . . 209
Nitrite of Amyl in Ague, . . . . . 213
Vehicles of Malaria, . . . . . 214
The First Insensibility from Ether, . . 214
Vinegar as a Post Partum Hemostatic, . 215

SOCIETY REPORTS:

Buffalo Medical Association, . . . . 216

EDITORIAL:

Professional Cowardice, . . . . . 220
Mortality Table, . . . . . . . . 222
Maltine, . . . . . . . . . . 222

REVIEWS, . . . . . . . 223
Books and Pamphlets Received, . . . . 228

